# Seaweed Supplementation Enhances Maximal Muscular Strength and Attenuates Resistance Exercise-Induced Oxidative Stress in Rats

**DOI:** 10.1155/2019/3528932

**Published:** 2019-07-28

**Authors:** Mallikarjuna Korivi, Chun-Tai Chen, Szu-Hsien Yu, Weibing Ye, I-Shiung Cheng, Jhong-Sin Chang, Chia-Hua Kuo, Chien-Wen Hou

**Affiliations:** ^1^Exercise and Metabolism Research Center, College of Physical Education and Health Sciences, Zhejiang Normal University, Jinhua 321004, China; ^2^Laboratory of Exercise Biochemistry, Institute of Sports Sciences, University of Taipei, Taipei 11153, Taiwan; ^3^Department of Leisure Industry and Health Promotion, National Ilan University, Yilan County 26047, Taiwan; ^4^Department of Physical Education, National Taichung University of Education, Taichung City 40306, Taiwan; ^5^Taiwan Fertilizer Co., LTD, Hualien 97064, Taiwan

## Abstract

We investigated the effect of chronic seaweed (*Gracilaria asiatica*) supplementation on maximal carrying capacity, muscle mass, and oxidative stress in rats following high-intensity resistance exercise (RE). Forty Sprague-Daley rats were equally categorized into control, exercise, seaweed, and exercise plus seaweed (ES) groups. Rats in respective groups performed RE (once per 2 days) or received seaweed (250 mg/kg bodyweight, orally) for 10 weeks. Results showed that seaweed consumption in combination with RE significantly (*p* < 0.05) increased maximal weight carrying capacity compared to RE alone. FHL muscle mass was significantly higher in both exercise and ES groups. Notably, high-intensity RE-induced lipid peroxidation, as evidenced by elevated thiobarbituric acid reactive substances (TBARS) in muscle, was substantially diminished (*p* < 0.05) by seaweed treatment. This antioxidative effect of seaweed was further represented by augmented superoxide dismutase activity and glutathione levels in seaweed groups. We noticed increased insulin concentrations and HOMA-IR, while the fasting blood glucose levels remained stable in seaweed and ES groups. Our findings conclude that seaweed in combination with RE enhanced maximal carrying strength and attenuated oxidative stress through improved antioxidant capacity. Seaweed could be a potential nutritional supplement to boost performance and to prevent exercise-induced muscle damage.

## 1. Introduction

Resistance exercise (RE) is the most effective lifestyle intervention to promote overall health, to reduce body fat, and to gain muscular strength. Greater muscular strength is strongly associated with improved athletic performance, and RE has been shown to be more effective in improving the muscular strength, muscle mass, and quality [1–3]. Multiple sets of RE can increase the muscle protein synthesis in rat skeletal muscle, considered to be a fundamental phenomenon in increasing the muscle mass [4, 5]. Besides, ingestion of nutrients (proteins/amino acids) combined with RE was reported to augment protein synthesis acutely and result in muscle hypertrophy over a period of time [6, 7]. Conversely, insufficient nutrients or longer fasting periods after RE promote muscle protein breakdown, which led to a state of negative net protein balance in muscle [4]. Thus, intake of sufficient nutrients following RE is paramount important when muscular hypertrophy or strength is the primary goal [6]. Indeed, muscle growth or tissue remodeling is modulated by several hormones, including insulin, which play a multifaceted role in skeletal muscle metabolism. Insulin was reported to reduce muscle protein breakdown; however, its action on protein synthesis is equivocal and depends on the nutritional status following exercise [8]. Taken together, exercise, insulin, and nutrients are implicated in muscle protein synthesis.

Despite the fact that RE can increase the muscle mass, some studies have shown increased oxidative stress after RE in both animals [9] and humans [10]. Oxidative stress is an imbalance between cellular antioxidant status and amount of free radicals or reactive oxygen species (ROS). Skeletal muscle is a potent site of ROS generation during contraction and accumulation of ROS in muscle represented by a decreased antioxidant status and increased oxidative stress. In this milieu, decreased superoxide dismutase (SOD), catalase, glutathione peroxidase (GPx), and glutathione (GSH) subsequently increase the oxidative damage to proteins, lipids, and DNA [11, 12]. It has been reported that high-intensity hypertrophy training (75-80% 1RM) and strength training (90-100% 1RM) increased superoxide anion (O_2_^•−^) production in rat skeletal muscle. This was accompanied by severely increased oxidative damage to lipids and proteins after hypertrophy training, but not with strength training [9]. The association between oxidative stress and exercise is said to be extremely complex and mostly depend on the type, intensity, and/or duration of exercise. In this context, intake of whole foods that contain antioxidants in natural ratios could be a practical strategy to prevent the RE-induced oxidative stress [13].

Consumption of marine seaweeds as food substance and their usage as a folk medicine to treat diseases have centuries of history in East Asian territories, including Taiwan, China, Japan, Korea, and Vietnam [14–16]. In recent four decades, the usage of edible seaweeds has increased in Western and developing counties, due to their rich nutritional values and pharmacological potentials [17, 18]. The genus* Gracilaria* (red algae), one of the notable and largest genera in* Gracilariaceae *family, is widely distributed in tropical and subtropical regions [19, 20].* Gracilaria* species are extensively used in food and pharmaceutical industries as they possesses various primary and secondary bioactive metabolites, such as carbohydrates, polyunsaturated fatty acids, dietary fibers, carotenoids, phycoerythrin, prostaglandin, sterols, proteoglycan, vitamins, and minerals [16, 21]. Recent studies explored the antitumor [22], antioxidant [23], antidiabetic [24], and anti-inflammatory [25] properties of bioactive metabolites of* Gracilaria*.

Although extensive research has been done on various* Gracilaria* species, only few reports are available on* Gracilaria asiatica *Zhang & Xia, one of the popular red alga in East Asia. For instance, Sajiki and Kakimi identified the major eicosanoids, including prostaglandin (PG) E_2_ from* G. asiatica* [26]. Another study isolated three novel compounds from* G. asiatica*, namely, gracilarioside and gracilamide A and B, bearing an unusual cyclopropane ring. All these compounds showed mild-to-weak cytotoxicity against human melanoma cells [27].* G. asiatica* is cultivated on a large scale in Vietnam and is the main seaweed for the production of agar.* G. asiatica* is composed of considerable amounts of chlorophyll, carotenoid, niacin, and vitamin C [16]. However, yet no experimental results are available to demonstrate the* in vivo* biological activities of* G. asiatica*. In view of the increased consumption of red algae, it is worth examining whether* G. asiatica *supplementation could cope with the oxidative stress and enhance exercise performance in rats. In this study, we investigated the effect of chronic* G. asiatica *supplementation on maximal carrying capacity and redox imbalance in rats after high-intensity progressive RE. Redox imbalance was evaluated by measuring the biomarkers of lipid peroxidation and antioxidant status.

## 2. Materials and Methods

### 2.1. Animals

Forty male Sprague-Dawley (SD) rats weighting 299.8±12.9 g were obtained from the BioLASCO Taiwan Co. Ltd., Taipei. Two rats per cage were housed in a temperature controlled animal facility center (22-24°C) with 12:12 h light-dark cycle and 50% humidity. All rats had free access to standard laboratory diet (LabDiet 5001; PMI Nutrition International, Brentwood, MO, USA) and water* ad libitum*. All experimental protocols used in this study were reviewed and approved (No. UT104003) by the Institutional Animal Care and Use Committee (IACUC) of University of Taipei. The entire study conformed to the Guidelines for the “Use of Research Animals” published by the Council of Agriculture, Executive Yuan, Taiwan.

### 2.2. Experiment Design

After one week of acclimatization to the laboratory conditions, rats were randomly assigned to 4 groups, 10 in each, including control (C), seaweed (S), exercise (E), and exercise plus seaweed (ES) groups. Rats in E and ES groups performed ladder-climbing resistance training every 2 days (alternate days) for a period of 10 weeks. Seaweed for respective groups was orally supplemented at the dose of 250 mg/kg bodyweight for 10 weeks, and rats in control group received equal amounts of saline. Daily food and water intake were recorded, and no statistical differences were found among the groups. After the last training session/treatment (48 h), all rats were sacrificed under anesthesia with an intraperitoneal injection of choral hydrate (400 mg/kg bodyweight). Then flexor hallucis longus (FHL) muscle samples were excised, weighted, immediately frozen in liquid nitrogen, and stored at −80°C until further biochemical assays.

### 2.3. Seaweed Cultivation and Nutritional Composition

Seaweed (*G. asiatica*) was provided by the Taiwan Yes Deep Ocean Water Co., Ltd. (Hualien, Taiwan). Seaweed cultivation was carried out in a 4-tonne FRP tank. Briefly, a PVC water pipe connection blower was installed at the bottom of the tank to pump the sea water vigorously into the tank. This arrangement provides sufficient light and nutrients to promote the growth of seaweed. The sea water supply continuously flowed into the tank to maintain the water quality and stable nutrients in water. During the cultivation (4 weeks) no additional nutrients were added to the water, and special care has been taken to maintain the stable temperature (18-22°C). The seaweed was collected, washed with fresh water, placed in the air-drying rack for 1 h, and then dried for 8 h at 60°C. The dried seaweed was tested for chemical composition, and results were presented in Table 1.

### 2.4. Resistance Exercise Training and Assessment of Maximal Strength

Rats in exercise groups performed ladder-climbing progressive resistance training according to the protocol developed by Hornberger and Farrar (2004) [28]. This mode of resistance training was introduced to mimic the human progressive RE training model. In particular, physiological adaptations and degree of hypertrophy occurring in rat FHL muscle were similar to those observed in human skeletal muscle [28]. Prior to the experiment, rats were familiarized for one week with climbing a vertical ladder (length 110 cm, grid 2 cm, 80° incline). During the training session, rats were motivated to climb from bottom to top of the ladder, and a housing cage was placed on the top to rest for 2 min. Rats were then placed at the bottom again and allowed to climb to the top of the ladder for 3 repetitions with 2 min interval. For the first training secession (after familiarization), load apparatus was secured to the bottom of the tail and then allowed to perform RE regimen as described above. The first training carrying load was 65% of the rat's bodyweight. Then 30 g of additional weight was added until rats are unable to climb the entire length of the ladder, and maximal carrying capacity was recorded. During subsequent 4 ladder climbs, rats carried respective 65%, 85%, 95%, and 100% of their previous maximal capacity. An additional 30 g load was added during subsequent climbs until exhaustion or reaching 8 repetitions, and maximal strength was recorded [29]. During whole 10-week RE regimen, rats performed a total of 35 sessions, and maximal carrying capacity was measured every time upon RE performance until the last (35^th^) RE session.

### 2.5. Assessment of Glucose, Insulin, and HOMA-IR

The effect of seaweed supplementation on fasting blood glucose, insulin, and HOMA-IR was evaluated at the end of the treatment. Blood samples were collected from the tail vein of rat after an overnight fasting (8 h). Fasting blood glucose levels were determined by glucose analyzer (Roche, Mannheim, Germany). Insulin concentrations were estimated using an enzyme-linked immunosorbent assay (ELISA) analyzer (Tecan Genios, Salzburg, Austria). For insulin assay, 200 *μ*L of blood sample was centrifuged at 3000 rpm of 10 min, and serum was collected into a separate tube. Insulin levels were then determined using ELISA kit provided by Diagnostic Systems Laboratories (Webster, TX, USA). HOMA-IR was calculated based on the fasting blood glucose and insulin values.

### 2.6. Lipid Peroxidation Assay

Lipid peroxidation in the muscle samples was determined by measuring the thiobarbituric acid reactive substances (TBARS). Malondialdehyde (MDA), a product of lipid peroxidation, combined with thiobarbituric acid (TBA) under high temperature and formed MDA-TBA adducts. According to the TBARS assay kit (Cayman Chemical Company, MI, USA), MDA-TBA adducts (colored compound) in the muscle samples were measured at 535 nm in a spectrophotometer (Tecan Genios, A-5082, Austria).

### 2.7. Determination of Superoxide Dismutase Activity

Muscle SOD activity was determined according to the protocol provided by Cayman's SOD assay kit (Ann Arbor, MI, USA), which utilizes tetrazolium salt for detection of superoxide radicals generated by xanthine oxidase and hypoxanthine. The absorbance of the reaction mixture was read at 450 nm using ELISA reader (Tecan Genios, A-5082, Austria). One unit of SOD activity was defined as the amount of enzyme needed to exhibit 50% dismutation of the superoxide radical. Final SOD activity in the sample was expressed as units per mg protein.

### 2.8. Estimation of Reduced Glutathione Content

The reduced form of glutathione (GSH) in the muscle samples was estimated using the kit provided by Cayman Chemical Company (Ann Arbor, MI, USA). GSH in the sample was combined with DTNB (5,5′-dithiobis-2-nitrobenzoic acid) and produced a colored product TNB (5-thio-2-nitrobenzoic acid). The concentration of TNB was measured at 415 nm by ELISA reader (Tecan Genios, A-5082, Austria).

### 2.9. Statistical Analyses

All values were expressed as mean ± standard error (SE) for ten replicates. One-way analysis of variance (ANOVA) was used to compare the effects of seaweed supplementation and RE on muscle parameters. SPSS and MS Office Excel were used to run the statistical analyses. Duncan's* post hoc* test was used to compare the significant differences between the groups. P value less than 0.05 was considered as statistical significance.

## 3. Results

### 3.1. Nutritional and Chemical Composition of Dried Seaweed Powder

The detailed chemical constituents that are present in dried seaweed powder were shown in Table 1. The dried* G. asiatica* powder possesses rich nutritional values and various trace elements. The nutritional composition includes 14.1 g of protein, 0.1 g of fat and 32.4 g of carbohydrate with 135.1 Kcal of energy per 100 g. In addition,* G. asiatica* contains 26.5 g/100 g of dietary fiber. The major elements of seaweed include potassium (395000 ppm), magnesium (9180 ppm), sodium (37100 ppm), and calcium (6510 ppm). Along with these, Zn, Ma, and Fe at 18.6, 9.29, and 114 ppm, respectively, were also found in seaweed powder.

### 3.2. Seaweed Plus RE Increases FHL Muscle Mass

**Figure 1:**
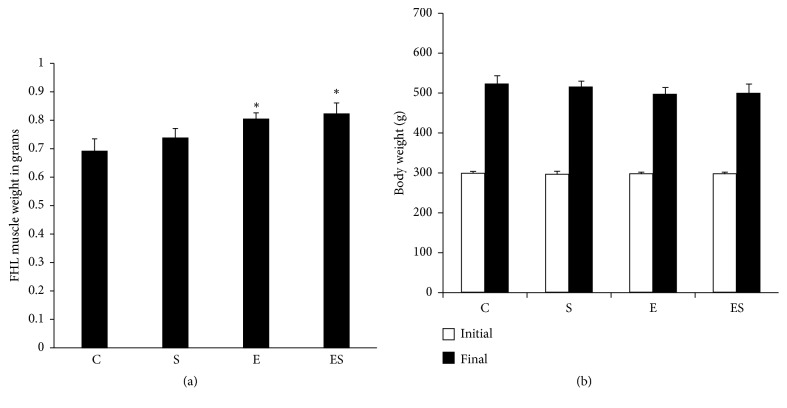
Seaweed plus resistance training increases FHL muscle mass. FHL muscle samples were weighted at the end of the study (a). Initial and final body weights for all groups were presented as histogram (b). Values are expressed as mean ± SE (*n* = 10). Results are significant compared with control (*p* < 0.05).

In this study, we found that high-intensity progressive RE alone and also in combination with seaweed supplementation significantly (*p* < 0.05) increased the absolute FHL muscle mass compared with the control (Figure 1(a)). The FHL muscle weight when presented relatively to bodyweight was also increased in E and ES groups. Conversely, no significant differences in bodyweight (Figure 1(b)) and food intake (data not shown) were noticed among the groups.

### 3.3. Effect of Seaweed Supplementation on Blood Glucose Insulin Concentrations

To test the effect of chronic seaweed ingestion on glucose homeostasis, we measured the fasting blood glucose and insulin levels in this study. We found no significant differences in fasting glucose levels with any treatment. However, fasting insulin levels were significantly (*p* < 0.05) elevated in seaweed alone and ES groups compared to control. Increased insulin concentrations appears to elevate the HOMA-IR values in seaweed (3.9±0.5) and exercise plus seaweed (5.1±1) groups than the control values (3±0.3) (Table 2). Despite the unchanged glucose levels, further investigations are essential to confirm the seaweed effect on whole body insulin sensitivity in rats.

### 3.4. Seaweed Enhances Maximal Carrying Capacity during Treatment Period

**Figure 2:**
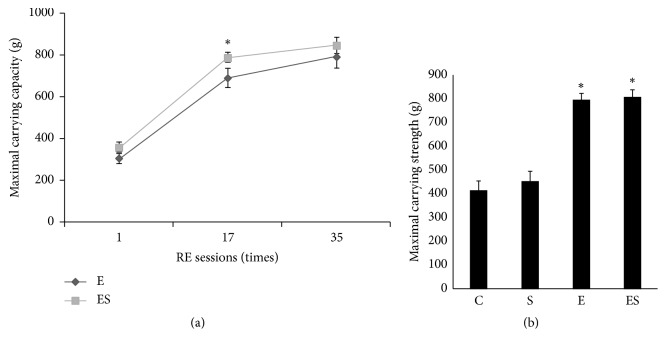
Seaweed combination enhances the maximal carrying capacity more than that of resistance exercise alone. Changes in weight carrying capacity (a) were recorded upon every RE session (35 times) during 10 weeks. *∗* indicates significant difference between exercise and exercise plus seaweed groups (*∗p* < 0.05). Maximal strength (b) was determined at the end of treatment. Results are significant compared with control (*∗p* < 0.05). All values are expressed as mean ± SE (*n* = 10).

One of the key statures of our study is that seaweed supplementation plus exercise training rapidly increased the maximal carrying capacity compared to exercise training alone. In a total of 35 RE sessions (10 weeks), increased weight carrying strength in ES group was noticed shortly after seaweed supplementation that continued almost up to 17th RE regimen (*p* < 0.05) and became plateau thereafter (Figure 2(a)). In addition, the final maximum strength estimated at the end of the treatment was significantly higher (*p* < 0.05) in exercise alone and also in exercise plus seaweed combination groups (Figure 2(b)).

### 3.5. Seaweed Supplementation Suppresses RE-Induced Lipid Peroxidation in Muscle

**Figure 3:**
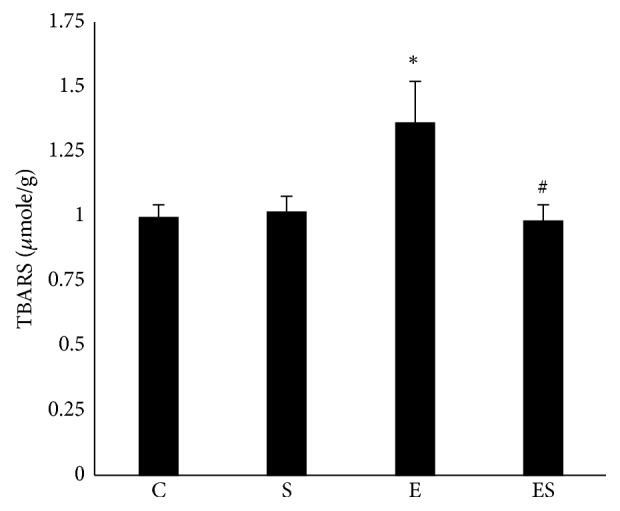
Seaweed supplementation diminishes resistance exercise-induced lipid peroxidation (TBARS) in muscle. Values are expressed as mean ± SE (*n* = 10). Results are significant compared with control (*∗p* < 0.05) and exercise (#*p* <0.05) groups.

To address the effect of chronic seaweed supplementation on muscle redox homeostasis, TBARS, a hallmark of lipid peroxidation was determined in this study. We found that high-intensity progressive RE remarkably increased the muscle lipid peroxidation, as evidenced by elevated (*p* < 0.05) TBARS. However, the elevated TBARS were substantially decreased (*p* < 0.05) when RE group combinedly treated with seaweed. The decreased RE-induced TBARS in ES group implies that seaweed is capable of quenching the RE-induced toxic ROS and thereby suppressing the lipid peroxidation (Figure 3).

### 3.6. Seaweed Supplementation Enhances SOD Activity against RE-Induced Stress

**Figure 4:**
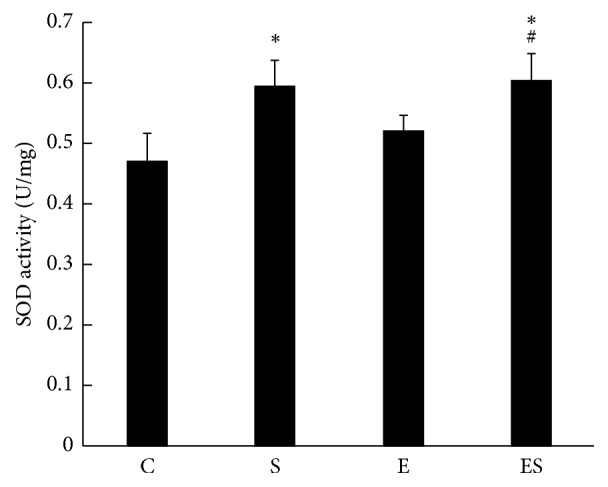
Seaweed supplementation increases muscle superoxide dismutase (SOD) activity against resistance exercise-induced stress. Values are expressed as mean ± SE (*n* = 10). Results are significant compared with control (*∗p* < 0.05) and exercise (#*p* <0.05) groups.

We then measured the SOD activity to demonstrate whether seaweed supplementation can promote muscle antioxidant capacity following high-intensity RE. We found that seaweed treatment alone significantly increased (*p* < 0.05) the SOD activity more than that of control. Seaweed combined with ladder-climbing exercise also augmented SOD activity, which is statistically significant compared with control and exercise groups. Our findings revealed that seaweed can promote tissue antioxidant status against high-intensity RE-induced oxidative stress (Figure 4).

### 3.7. Seaweed Plus Exercise Combinedly Preserves Glutathione Homeostasis

**Figure 5:**
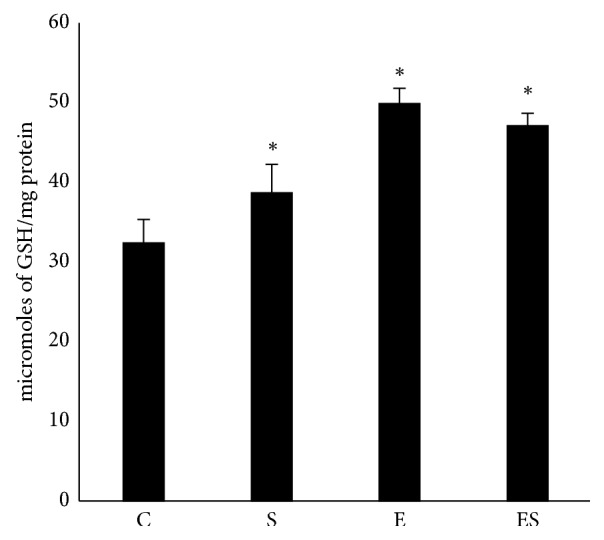
Seaweed combined with resistance exercise training preserves muscle glutathione levels. Values are expressed as mean ± SE (*n* = 10). Results are significant compared with control (*∗p* < 0.05).

Glutathione homeostasis is crucial to counteract the ROS-induced oxidative damage [30]. In harmony with increased SOD activity, we found seaweed supplementation notably increased the GSH content in FHL muscle (*p* < 0.05). Moreover, exercise training alone and in combination with seaweed also promoted GSH levels (Figure 5). Taken together, chronic seaweed supplementation appears to be beneficial in maintaining of the intracellular redox homeostasis.

## 4. Discussion

Our findings primarily demonstrated that chronic seaweed (*G. asiatica*) supplementation could attenuate RE-induced oxidative stress, increase HFL muscle mass, and enhance maximal carrying capacity when combinedly treated with high-intensity progressive RE. Compared to RE alone, combination of seaweed and RE intensifies the maximal carrying strength shortly after consumption, and this trend was continued almost up to 5^th^ week (17^th^ RE session). In addition, we found that RE-induced lipid peroxidation in HFL muscle was significantly inhibited by seaweed. This protective effect was revealed by a substantial decrease of TBARS. Notably, seaweed ingestion alone is capable of promoting the muscle SOD activity and GSH levels. Increased antioxidant status with seaweed perhaps contributed to decrease the RE-induced lipid peroxidation. Conversely, 10-week seaweed consumption appears to elevate serum insulin concentrations, while fasting blood glucose levels remained stable.

The beneficial effects of RE are said to be maximized by three basic principles; of those “progressive overload” is the primary one. Progressive overload is the gradual increase of stress/weight placed on the body during RE performance [2]. In our study, we employed high-intensity progressive RE protocol (ladder-climbing) and found increased FHL muscle mass and maximal weight carrying capacity of rats after 10 weeks. Ladder-climbing resistance training with progressive overloads has been demonstrated to increase muscular strength or maximal weight carrying capacity of rats even after 8 weeks [28, 31, 32]. The key finding of our study is that seaweed in combination with RE further enhances the weight carrying strength. Most importantly, weight carrying capacity was greater in seaweed plus RE group shortly after seaweed intake and continued up to 17th RE session. According to our knowledge, this is the first report to demonstrate that* G. asiatica* ingestion enhanced maximal carrying capacity in rats. Although there is no molecular evidence to support this phenomenon, we assume that chemical constituents and nutritional values existing in seaweed may be contributed to the increased maximal carrying strength and FHL muscle mass.

Skeletal muscle mass is basically regulated by the firm balance of muscle protein synthesis and muscle protein breakdown. Multiple sets of progressive RE are known to increase muscle protein synthesis, muscle mass, and muscle quality [2, 4–6]. The increased FHL muscle mass in our study after ladder-climbing RE was in agreement with Hornberger and Farrar findings, who reported FHL hypertrophy after progressive weight loading training. Moreover, training adaptations, such as contractile properties and hypertrophy, occurring in rat FHL muscle were similar to those identified in human skeletal muscle following RE [28]. FHL muscles are predominantly fast-twitch glycolytic (type IIB) fibers [33, 34] recruited during high-intensity burst, such as maximal lifts. The absolute increase in FHL muscle mass with RE plus seaweed ingestion may be attributed to the enhancement in the maximal weight carrying capacity of rats. Together with RE, supplementation of nutrients (amino acids, proteins) was reported to increase muscle protein synthesis acutely, which then contributed to hypertrophy over a period of time [6, 7], whereas longer fasting periods or insufficient availability of nutrients following RE regimen can cause muscle protein breakdown, which renders negative net protein balance in skeletal muscle [4]. Therefore, to gain the greater benefits of progressive RE, it is important to provide enough nutrients following RE. Seaweed supplementation along with RE in our study could supply the nutrients demand and thereby increase maximal carrying strength and muscle mass.

Literature revealed that exercise stimulus and nutritional interventions are implicated in skeletal muscle protein synthesis and increase muscle mass. In addition, insulin also plays a multifaceted role in skeletal muscle metabolism and growth. Despite the arguable action of insulin on muscle protein synthesis, it is capable of decreasing the muscle protein breakdown following exercise [8, 35]. Studies have claimed that ingestion of lower-dose of proteins with carbohydrates may influence the muscle net protein balance possibly through the increased insulin concentrations, inhibited muscle protein breakdown, and/or increased amino acid distribution to the muscle tissue [4]. In our study, supplementation of seaweed that consisted of proteins and carbohydrates also resulted in increased insulin levels. Interestingly, the increased insulin was accompanied by an increased muscle mass and maximal strength. Taken together, higher insulin levels in seaweed plus exercise group may be at least in part responsible for the rapid increase of maximal carrying capacity and muscle mass, which needs to be confirmed. Regardless of insulin response to treatment combination, the unchanged blood glucose levels imply the stable glucose homeostasis in rats. We speculate that RE-induced oxidative stress, which was also seen in our study, may instigate in incidence of such insulin response. Of note, accumulation of ROS can lead to abnormal changes in intracellular signaling and cause insulin resistance. Molecular basis of oxidative stress-induced insulin resistance has been attributed to ROS triggering the activation of serine/threonine kinase cascades (NF-*κ*B, JNK, MAPK) which phosphorylate insulin receptor and insulin receptor substrate [36]. So far there is no direct evidence to demonstrate that* G. asiatica* deteriorates insulin sensitivity in animals or humans. Therefore, our findings offering further in-depth investigations on the effect of* G. asiatica* and insulin response in RE rats. The dosage and duration of* G. asiatica* treatment and involved signaling cascades in insulin action need to be thoroughly studied.

Skeletal muscle cells are the potential sources of ROS production during resistance exercise due to repetitive contractions of muscles. High-force eccentric exercise triggers several pathways, including activation of NADPH oxidase, xanthine oxidase, and calcium overload that are attributed to excessive ROS production and muscle injury [37, 38]. Accumulation of intracellular ROS from high-intensity RE or exhaustive exercise eventually causes oxidative damage to lipids, proteins, and DNA molecules. In lipid peroxidation process, ROS rapidly attack the unsaturated fatty acid residues of phospholipids in cell membrane, which results in loss of membrane integrity. The higher levels of oxidative stress further increase the production of several peroxides and aldehydes that are toxic to muscle cells [39]. In our study, muscle TBARS (lipid peroxidation byproducts) were multiplied with progressive RE, which indicates increased oxidative damage to membrane lipids. One of the key finding of our study is that seaweed supplementation decreased the elevated TBARS, which means diminished lipid peroxidation against RE. Seaweed was reported to contain considerable amounts of minerals, phytochemicals, polyphenols, vitamin E, and carotenoids, which could act as antioxidants and thereby reduce hydroxyl radical and superoxide anion-induced lipid peroxidation and LDL oxidation [40]. In this regard, a study showed that supplementation of dried seaweed powder inhibited high-fat diet-induced lipid peroxidation in rats [41]. Furthermore, pretreatment of red algae (*G. caudate*) polysaccharides was reported to decrease the gastric MDA levels against alcohol-induced elevation, and this protective effect was associated with increased antioxidant status in alcohol plus seaweed fed mice [42]. In our study, seaweed-mediated inhibition TBARS against high-intensity RE were assumed to be associated with increased antioxidant status or ROS scavenging activity.

Among several antioxidant enzymes, SOD is the first and most important enzyme that scavenges toxic O_2_^•−^ radicals into less toxic H_2_O_2_ [43, 44]. A previous study demonstrated that 12-week muscular resistance training (~65% 1RM) had no effect on either O_2_^•−^ production or SOD activity, but high-intensity hypertrophy training (75%-80%1RM) and strength training (90%-100%1RM) increased O_2_^•−^ production and SOD activity in rat skeletal muscle [9]. In our study, high-intensity RE alone had no effect on muscle SOD; however, seaweed treatment alone and also in combination with RE augmented SOD activity. Increased SOD might be responsible for the effective reduction of RE-induced lipid peroxidation. Chronic seaweed (5% and 10%) supplementation for 8 weeks was reported to increase SOD activity in rats fed normal diet, but not high-fat diet [41]. A recent study showed that polysaccharides of seaweeds effectively scavenged O_2_^•−^ radicals and suppressed lipid peroxidation [45].* Gracilaria* species contain much higher fiber content (~20%) than the well-known antioxidant vegetables, such as broccoli, carrot, and pumpkin [46]. In particular,* G. asiatica* possesses rich amounts of various macro- and microelements along with carotenoids, vitamin C, vitamin A, and niacin [16]. We also reported both macroelements (K, Na, Mg, Ca) and microelements (Zn, Mn, Fe, B) in* G. asiatica* powder. In addition to the considerable amounts of zinc and manganese,* G. asiatica* also possesses rich quantity of dietary fiber. Since endogenous SOD activity is associated with bioavailability of copper/zinc, manganese, or other nutrients [44], consumption of* G. asiatica* is postulated to enhance the bioavailability of these elements and thereby increase SOD activity to diminish oxidative stress.

GSH, a foot marker of oxidative stress, plays a vital role in defense mechanism and regulates the intracellular redox homeostasis [43, 47]. We found increased muscle GSH with seaweed, RE, and combination of both. Increased GSH content indicates the antioxidant property of seaweed, while it might be a self-protective mechanism against high-intensity RE. It has been shown that physical exercise or muscle contractile activity increases cellular ROS production and imposes oxidative stress [12]. Hence, it is possible that continuous exposure of muscle cells to such oxidative insult may necessarily promote cellular defensive mechanism as an adaptive response. In this milieu, both acute and chronic exercises have been reported to enhance GSH levels in rat skeletal muscle. Alternatively, exercise also activates important redox-sensitive proteins (NF-*κ*B, Nrf2, PGC-1*α*, JNK) that are likely to be implicated in modulation of such essential adaptation [12, 43]. Seaweed extracts were reported to improve GSH levels in the gastric mucosa of mice and prevent alcohol-induced lipid peroxidation and gastric damage [42]. Vitamin A, vitamin C, and niacin in* G. asiatica* [16] along with other essential elements possibly felicitate cellular redox homeostasis and GSH stability in muscle cells. Besides, previous studies reported that* Gracilaria* species can synthesize polysaccharides, which are primarily responsible for the antioxidant properties [23, 42, 45]. In this pharmacological point of view, seaweed supplementation appears to be effective in improving the tissue antioxidant status.

## 5. Conclusions

For the first time, we demonstrated that* G. asiatica* supplementation in combination with high-intensity RE significantly increased maximal carrying capacity and FHL muscle mass in rats. Compared to RE alone, weight carrying strength in combination group was greater shortly after seaweed consumption. Besides, seaweed supplementation considerably diminished RE-induced lipid peroxidation in skeletal muscle. Decreased lipid peroxidation was attributed to increased antioxidant status (SOD activity and GSH content) in RE plus seaweed treated group. Our findings suggest that seaweed consumption as a complimentary medicine might be beneficial in coping with RE-induced oxidative insult and improving weight carrying capacity in rats.

## Figures and Tables

**Table 1:** Nutritional composition and elements of dried seaweed powder.

Nutrients/elements	Quantity	Unit
Protein	14.4	g/100g
Fat	0.1	g/100g
Carbohydrate	32.4	g/100g
Dietary fiber	26.5	g/100g
Na	37100	ppm
B	166	ppm
Fe	114	ppm
K	395000	ppm
Mg	9180	ppm
Sr	84.1	ppm
Ca	6510	ppm
Zn	18.6	ppm
Ni	3.96	ppm
Se	0.11	ppm
Mn	9.29	ppm

**Table 2:** Effect of seaweed supplementation on glycemic control in rats. Fasting blood glucose and insulin levels were determined after 10-week treatment, and HOMA-IR was calculated. Values are expressed as mean ± SE (*n* = 10). Results are significant compared with control (*∗p* < 0.05) and seaweed (†*p* <0.05) groups.

	Groups			
	Control	Exercise	Seaweed	Exercise + Seaweed
Glucose (mg/dL)	101.2 ± 4	102.8 ± 4.1	102.8 ± 3.1	101.8 ± 4.6
Insulin (uU/L)	12.0 ± 1	11.7 ± 1.8	15.2 ± 1.9*∗*	19.4 ± 3.1*∗*†
HOMA-IR	3±0.3	3.1±0.6	3.9±0.5*∗*	5.1±1*∗*†

## Data Availability

The data used to support the findings of this study are available from the corresponding author upon request.
